# Biomechanical evaluation of a new pedicle screw-based posterior dynamic stabilization device (Awesome Rod System) - a finite element analysis

**DOI:** 10.1186/s12891-015-0538-x

**Published:** 2015-04-09

**Authors:** Chen-Sheng Chen, Chang-Hung Huang, Shih-Liang Shih

**Affiliations:** Department of Physical Therapy and Assistive Technology, National Yang-Ming University, Taipei, Taiwan; Biomedical Research, Mackay Memorial Hospital, Tamshui District, New Taipei City, Taiwan; Department of Orthopaedic Surgery, Zhong-Xing Branch of Taipei-City Hospital, No. 145, Zhengzhou Rd., Datong Dist., Taipei, 10341 Taiwan; Institute of Neuroscience, National Chengchi University, Taipei, Taiwan

**Keywords:** Posterior dynamic stabilization, Adjunct to fusion, Finite element model, Lumbar spine

## Abstract

**Background:**

Pedicle-screw-based posterior dynamic stabilization devices are designed to alleviate the rate of accelerated degeneration of the vertebral level adjacent to the level of spinal fusion. A new pedicle- screw-based posterior dynamic stabilization device- the Awesome Dynamic Rod System was designed with curve cuts on the rods to provide flexibility. The current study was conducted to evaluate the biomechanical properties of this new device.

**Methods:**

Finite element models were developed for the intact spine (INT), the Awesome Dynamic Rod Implanted at L4-L5 (AWE), a traditional rigid rod system implanted at L4-L5 along with an interbody cage (FUS), and the Awesome Dynamic Rod System implanted at L4-L5 along with an interbody cage as an adjunct to fusion procedures and extension of dynamic fixation to L3-L4 (AWEFUS). The models were subjected to axial loads and pure moments and evaluated by a hybrid method on range of motion (ROM)s, disc stresses, pedicle screws stresses, and facet joint contact forces.

**Results:**

FUS sustained the lowest L4-L5 ROM decrement in flexion and torsion. AWE demonstrated the lowest adjacent level ROM increment in all moments except for extension at L3-L4, and AWEFUS showed the greatest ROM increment at L2-L3. AWE demonstrated lowest adjacent segment disc stress in flexion, lateral bending and torsion at L3-L4. AWEFUS showed the highest disc stress increment in flexion, extension, and lateral bending, and the lowest disc stress decrement in torsion at L2-L3. AWE sustained greater adjacent facet joint contact forces than did FUS in extension and lateral bending at L3-L4, and AWEFUS demonstrated the greatest contact forces concentrating at L2-L3.

**Conclusion:**

The results demonstrate that the Awesome Dynamic Rod System preserved more bridged segment motion than did the traditional rigid rod fixation system except in extension. However, the Awesome Dynamic Rod System bore a greater facet joint contact force in extension. The Awesome Dynamic Rod System did protect the adjacent level of fusion segments, but led to much greater ROM, disc stresses, and facet joint contact forces increasing at the adjacent level of instrumented segments.

## Background

Spinal arthrodesis using a pedicle screw rigid rod system has been the standard treatment for degenerative lumbar diseases for several decades. In spite of the successful fusion rate and immediately satisfactory clinical results yielded, late sequelae relative to spinal arthrodesis has been reported, including accelerated degeneration of the vertebral level adjacent to the level of spinal fusion or instrumented segments (adjacent level) [1-4].

To alleviate the rate of accelerated degeneration of the adjacent level, motion preservation devices are designed to maintain mobility at the instrumented level. Among these devices, the pedicle-screw-based posterior dynamic stabilization (PDS) system is based on the principles of traditional pedicle-screw-based rigid rod systems for spinal fusion. The PDS system is amenable to use by surgeons and preserving the integrity of posterior ligaments and facet joints [5,6]. With the inherent characteristics of intervertebral motion preserved, the pedicle-screw-based PDS could be used as an adjunct to spinal fusion to facilitate graft fusing [7-9], or as a stand-alone system for non-fusion procedures [10-13]. Moreover, Gillet et al. [14] proposed extending dynamic fixation one level above the fusion segments to palliate the development of accelerated degeneration at the adjacent level. A finite element (FE) study conducted by Cheng et al. [15] proved the “preventative reinforcement” could eliminate the possibility of accelerated degeneration at the adjacent level, and clinical results pertaining to the minimization of degeneration at the adjacent level have been reported [16]. However, to date, there is no strong evidence to show that the pedicle-screw-based PDS eliminates the incidence of degeneration at the level adjacent to fusion or instrumented segments.

The Awesome Dynamic Rod System (New Taipei City, Baui Biotech, Co., Ltd., Taiwan), a new pedicle-based PDS system, is composed of traditional conical titanium pedicle screws and novel signed flexible rods. Instead of helical curve cuts on the rods, there are unique double curve cuts on the surface of sections of rods with enlarged diameters called “joint parts” that can produce small and limited vacant spaces to make the rods flexible to provide adequate movement of the spine in flexion-extension mode but not jeopardize stability.

The hypothesis of this current study is that as a type of pedicle-screw-based PDS system, the Awesome Rod Dynamic System can not only be used as a stand-alone system for dynamic fixation to preserve joint motion at the instrumented level, but it can also be used as an adjunct to fusion procedures and as a preventative reinforcement to alleviate the biomechanical effects on the adjacent level. A FE study using three-dimensional spinal models implanted with the Awesome Dynamic Rod System was designed to test this new pedicle-screw-based PDS system, and the corresponding range of motion (ROM), disc stress, facet joint contact forces of the spinal models, and pedicle screw stress of the Awesome Dynamic Rod System were calculated to evaluate the biomechanical effects of this device on the spinal column.

## Methods

### FE models of the lumbar spine and implants

**Figure 1:**
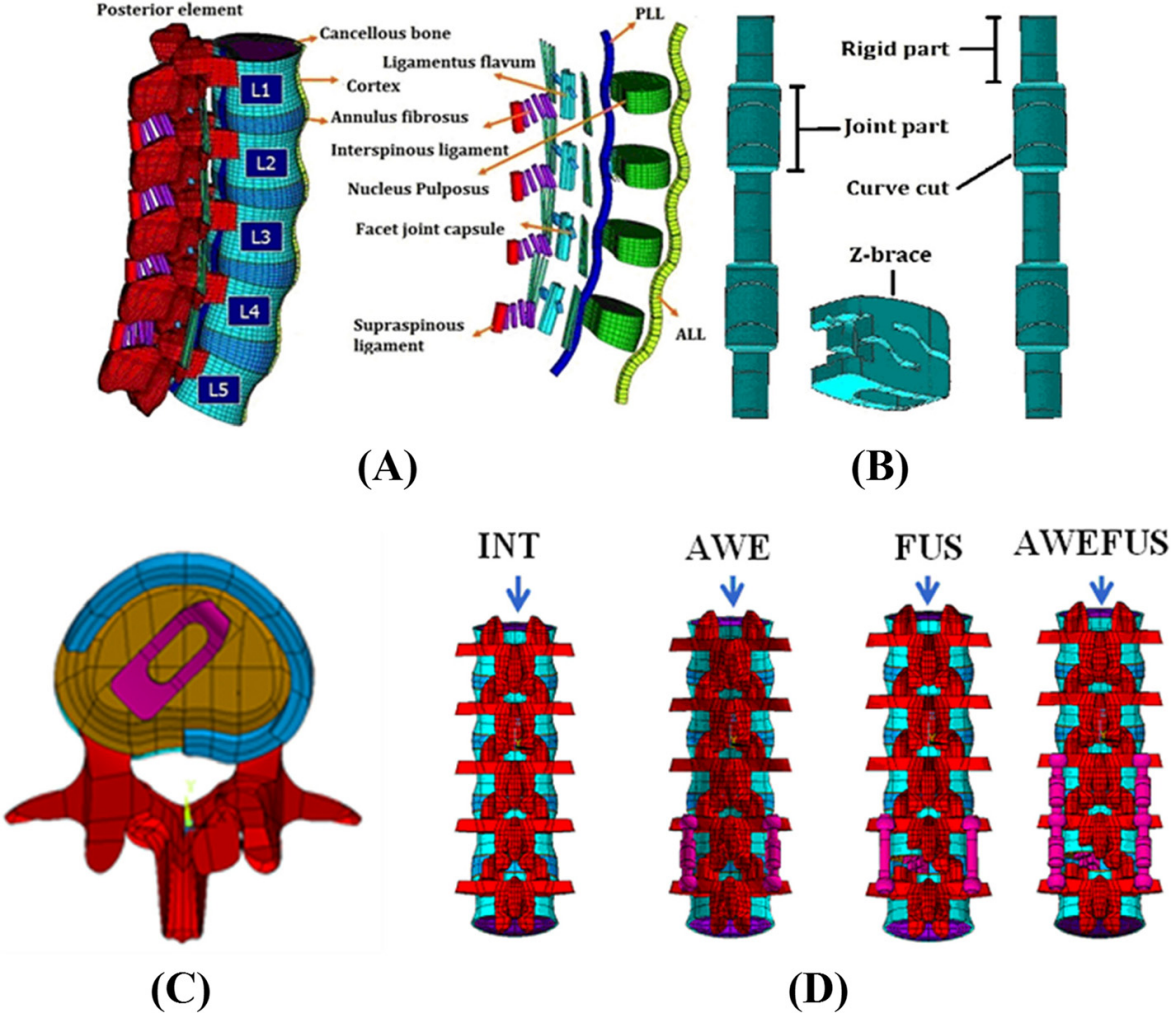
**Spine and implant FE models used in this study. (A)**. The osseous structures, intervertebral discs, and ligaments of the intact spine. **(B)**. The Awesome Dynamic Rod and Z-brace cage. **(C)**. At the L4-L5 disc space, the Z-brace cage was placed obliquely with the left posterolateral corner of the annulus fibrosus removed as in PLIF procedures. **(D)**. Four FE models used in this study.

A three-dimensional FE spine model was used in this study. The FE spinal model, which was constructed by ANSYS 11.0 (ANSYS Inc., Canonsburg, PA) consisted of osseoligamentous L1–L5 vertebrae, intervertebral discs, endplates, posterior bony elements, and all 7 ligaments as shown in Figure 1(A). The intervertebral disc consisted of an annulus fibrosus and nucleus pulposus, with 12 double cross-linked fiber layers embedded in the ground substance. The annulus ground substance was modeled based on an incompressible, hyperelastic, 2-parameter (C1, C2) Mooney-Rivlin formulation, and the nucleus pulposus was modeled as an incompressible fluid. Model construction and validation have been well documented in previous studies [17,18].

The dynamic rod FE models were stimulated with an Awesome Rod system consisting of conical titanium alloy screws and novel titanium rods, which were consisted of rigid part (diameter 5.5 mm) and joint part (diameter 6.0 mm with double curve cuts on the surface), as shown in Figure 1(B). The joint parts measured 10 mm in length and a 10 mm interval was established between every pair of joint parts. The uniquely designed joint parts provide flexibility that allows for approximately 10 degrees of dynamic motion. The interbody fusion device model Z-Brace cage (New Taipei City, Baui Biotech, Co., Ltd., Taiwan) is made from titanium alloy. The hollow device features deep slots on the upper and lower layers of the transverse plane, which produces a “Z” shape in the sagittal plane and provides 1 to 2 mm of dynamic compression.

The pedicle screws, standard rigid rods, Awesome Dynamic Rods, and Z-brace cages were modeled using 8-node solid elements. The titanium Z-Brace cage was implanted through the posterior lateral oblique approach obliquely across the coronal midline with appropriately sized. The left posterolateral corner of the L4-L5 annulus fibrosus was also removed to stimulate the status after posterior lateral interbody fusion procedures were performed [19] as shown in Figure 1(C).

To demonstrate the flexibility provided by the Awesome Dynamic Rod System, the following models were designed in this study: (1) intact spine (INT) without any implants, (2) spine implanted with pedicle screws and the Awesome Rod System at the L4-L5 level for dynamic fixation (AWE), (3) spine implanted with a Z-Brace cage and pedicle screws with a rigid rod system at L4-L5 as a fusion procedure (FUS), (4) spine implanted with a Z-Brace cage and the Awesome Dynamic Rod System at L4-L5 as an adjunct to fusion procedures and extension of the Awesome Dynamic Rod System to L3-L4 for dynamic fixation (AWEFUS) (Figure 1(D)). The interfaces between facet articular surfaces were treated as standard contact pairs at all levels. This study aimed to investigate the biomechanical effects on motion segment adjacent to the levels (L4-L5 at FUS and AWEFUS) already achieving bony fusion, thus, the interfaces between the Z-Brace cage and vertebral end plates, pedicle screws and vertebrae, pedicle screws and standard rigid rods, pedicle screws and Awesome Dynamic Rods were treated as bonded. The spinal fusion segments refer to the disc levels bridged with pedicle screws and rods combined with the Z-Brace cage. The instrumented segments refer to the vertebrae implanted with the Awesome Dynamic Rod System only, without the Z-Braces cage implanted within disc spaces. The vertebral level adjacent to the level of spinal fusion refers to L3-4 for FUS and AWEFUS, whereas the vertebral level adjacent to the level of instrumentations of the spine refers to L3-4 for AWE and L2-3 for AWEFUS.

### Boundary and loading condition

The lumbar spine FE models were fixed at the base of the fifth vertebrae. The hybrid method demonstrated by Panjabi was used to evaluate the effects on the adjacent spinal level [20]. Loads were applied on the models in two steps. In the first load step, an axial load of 150 N was applied perpendicular to the top of the L1 vertebrae. In the second load step, a pure unconstrained moment was applied in 0.36 Nm increments to ensure the resultant ROM (L1-L5) of all FE models would equal the ROM corresponding to 16 degrees in flexion, 9 degrees in extension, 17 degrees in left torsion, and 22 degrees in left lateral bending. The resultant ROM at the level adjacent to instrumentation/fusion, the instrumented level, and the total lumbar spine levels and the segment stiffness at the instrumented levels for each model are listed in Table 1.

**Table 1:** **ROM of four FE models at all motion segments**

**Motion**	**Model**	**L1-L2 (Degree)**	**L2-L3 (Degree)**	**L3-L4 (Degree)**	**L4-L5 (Degree)**	**Moment (Nm)**	**L1-L5 stiffness (Nm/Degree)**
Flexion	INT	3.63 (100%)	3.74 (100%)	3.71 (100%)	5.22 (100%)	3.8 (100%)	0.23 (100%)
	AWE	4.85 (134%)	4.79 (128%)	4.72 (127%)	1.84 (35%)	8.4 (221%)	0.52 (226%)
	FUS	4.85 (134%)	4.86 (130%)	5.8 (156%)	0.76 (15%)	8.4 (221%)	0.52 (226%)
	AWEFUS	6.85 (189%)	6.93 (185%)	1.62 (44%)	0.89 (17%)	13.65 (359%)	0.84 (365%)
Extension	INT	2.62 (100%)	2.23 (100%)	2.07 (100%)	2.01 (100%)	6.4 (100%)	0.72 (100%)
	AWE	3.18 (121%)	2.73 (122%)	2.7 (130%)	0.29 (14%)	8.05 (126%)	0.9 (125%)
	FUS	3.08 (118%)	2.63 (118%)	2.57 (124%)	0.55 (27%)	7.7 (120%)	0.87 (121%)
	AWEFUS	4.14 (158%)	3.56 (160%)	0.5 (24%)	0.74 (37%)	11.55 (180%)	1.29 (179%)
Lateral Bending	INT	7.11 (100%)	5.2 (100%)	5.07 (100%)	5.01 (100%)	8.2 (100%)	0.37 (100%)
	AWE	9.42 (132%)	6.18 (119%)	5.73 (113%)	1.18 (24%)	11.9 (145%)	0.53 (143%)
	FUS	9.44 (133%)	6.21 (119%)	5.79 (114%)	0.94 (19%)	11.9 (145%)	0.53 (143%)
	AWEFUS	12.5 (176%)	8.03 (154%)	1.22 (24%)	0.56 (11%)	15.75 (192%)	0.71 (192%)
Torsion	INT	5.58 (100%)	2.9 (100%)	3.4 (100%)	4.74 (100%)	9.7 (100%)	0.58 (100%)
	AWE	7.86 (141%)	3.14 (108%)	3.43 (101%)	2.18 (46%)	15.05 (155%)	0.91 (157%)
	FUS	8.08 (145%)	3.21 (111%)	3.5 (103%)	1.84 (39%)	15.4 (159%)	0.93 (160%)
	AWEFUS	9.5 (170%)	3.35 (116%)	1.79 (53%)	2.03 (43%)	17.85 (184%)	1.07 (184%)

The percentages indicate the ROM of all models normalized by the ROM of INT.

In this study, data pertaining to the ROM at each motion segment, the facet contact forces (FCFs) of L1-L5, and the peak disc stresses at L2-3 and L3-4 under flexion, extension, left torsion, and left lateral bending for all four models were gathered by the FE software, and these results are presented in Tables 1, 2 and 3 as percentages $\left(\frac{\mathrm{AWE}\ \mathrm{or}\ \mathrm{F}\mathrm{U}\mathrm{S}\ \mathrm{or}\ \mathrm{AWEFUS}}{\mathrm{INT}}\right)\times 100\%$.

**Table 2:** **Disc stresses at cephalic adjacent levels**

**Motion**	**Model**	**L2-L3 (KPa)**	**L3-L4 (KPa)**
Flexion	INT	621 (100%)	558 (100%)
	AWE	906 (146%)	782 (140%)
	FUS	915 (147%)	944 (169%)
	AWEFUS	1370 (221%)	661 (118%)
Extension	INT	399 (100%)	402 (100%)
	AWE	422 (106%)	455 (113%)
	FUS	410 (103%)	437 (109%)
	AWEFUS	525 (131%)	184 (46%)
Lateral Bending	INT	1050 (100%)	1040 (100%)
	AWE	1170 (111%)	1090 (105%)
	FUS	1180 (112%)	1090 (105%)
	AWEFUS	1840 (175%)	405 (39%)
Torsion	INT	540 (100%)	593 (100%)
	AWE	419 (78%)	441 (74%)
	FUS	436 (81%)	454 (77%)
	AWEFUS	488 (90%)	322 (54%)

The percentages indicate the disc stresses of all models normalized by the disc stresses of INT.

**Table 3:** **Facet joint forces in instrumented levels and cephalic adjacent levels**

**Motion**	**Model**	**L2-L3 (N)**	**L3-L4 (N)**	**L4-L5**
Extension	INT	108 (100%)	118 (100%)	108 (100%)
	AWE	138 (128%)	152 (129%)	0 (0%)
	FUS	132 (122%)	145 (123%)	0 (0%)
	AWEFUS	208 (193%)	0 (0%)	0 (0%)
Lateral Bending	INT	40 (100%)	15 (100%)	21 (100%)
	AWE	51 (128%)	44 (293%)	0 (0%)
	FUS	54 (135%)	44 (293%)	0 (0%)
	AWEFUS	110 (275%)	0 (0%)	0 (0%)
Torsion	INT	141 (10%)	143 (100%)	135 (100%)
	AWE	196 (139%)	204 (143%)	3 (2%)
	FUS	203 (144%)	210 (147%)	19 (14%)
	AWEFUS	259 (184%)	6 (4%)	28 (21%)

The percentages indicate the facet joint forces of all models normalized by the facet joint forces of INT.

## Results

### ROM at instrumented level

At the instrumented levels (L4-L5), the ROM of AWE, FUS, and AWEFUS decreased by 64.75%, 85.44%, and 82.95%, respectively, compared to that of INT during flexion; decreased by 85.57%, 72.64%, and 63.18%, respectively, compared to that of INT during extension; decreased by 76.45%, 81.24%, and 88.82%, respectively, compared to that of INT during left lateral bending; and decreased by 54.01%, 61.18%, and 57.17%, respectively, compared to that of INT during left torsion.

At the adjacent levels (L3-L4 for AWE and FUS but L2-L3 for AWEFUS), the ROM of AWE, FUS, and AWEFUS increased by 27.22%, 56.33%, and 85.29% in flexion; by 30.43%, 24.15%, and 59.64% in extension; by 13.02%, 14.20% and 54.42% in left lateral bending; and by 0.88%, 2.94%, and 15.52% in left torsion, respectively, compared to that of INT (Table 1).

### Disc stress at adjacent level

**Figure 2:**
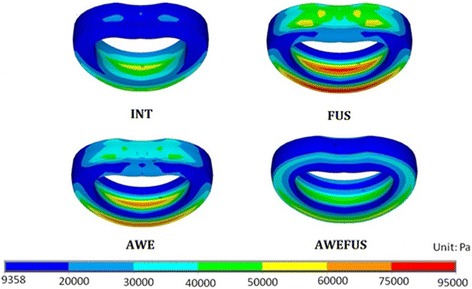
**Comparison of disc annulus stress at the adjacent level L3-L4 in flexion.** The maximum stress occurred at anterior edge of the annulus fibrosus. FUS showed the greatest stress. AWEFUS showed the lowest stress because levels L3-L4 were shielded by the Awesome Dynamic Rod System. AWE showed lower annulus stress than did FUS owing to its preservation of motion, which alleviated the effect of the adjacent level.

During flexion, the disc stress of AWE, FUS, and AWEFUS increased by 40.08%, 69.09%, and 120.70%, respectively, compared to that of INT. As shown in Figure 2, FUS exhibited higher stress at L3-L4 than did AWE in flexion. The maximum stress occurred at the anterior edge of the annulus fibrosus. FUS showed the greatest stress. AWEFUS showed the lowest stress because levels of L3-L4 were shielded by the Awesome Dynamic Rod System. AWE showed a lower annulus stress than did FUS owing to its preservation of motion, which alleviated the effect of the adjacent. During extension, the disc stress of AWE, FUS, and AWEFUS increased by 13.29%, 8.81%, and 31.46%, respectively, compared to that of INT. During left lateral bending, the disc stress of AWE, FUS, and AWEFUS increased by 4.81%, 4.81%, and 75.24%, respectively, compared to that of INT. However, during left torsion, the disc stress of AWE, FUS, and AWEFUS decreased by 25.69%, 23.50%, and 9.68%, respectively, compared to that of INT (Table 2).

### Facet contact forces at adjacent level

There was no facet contact force at any adjacent facet joint in flexion or left side facet joint in left torsion. Both facet contact forces of AWE, FUS, and AWEFUS increased by 28.81%, 22.03%, and 92.59%, respectively, compared to that of INT in extension. In left bending, the left side facet contact force of AWE, FUS, and AWEFUS increased by 113.33%, 106.67%, and 180.77%, respectively, whereas the right side facet contact force of AWEFUS (37 N) increased by 164.29% compared to that of INT. However, there was no facet contact force detected at right L3-4 facet joint of INT in left bending. The right side facet contact forces at L3-4 of AWE and FUS were 12 and 13 N, respectively, which were lower than the right side facet contact force at the adjacent level (L2-3) of AWEFUS (37 N). The right side facet contact force of AWE, FUS, and AWEFUS increased by 42.66%, 46.85%, and 83.69%, respectively, compared to that of INT in left torsion (Table 3).

## Discussion

Acting as a tension band at the posterior spinal column, the pedicle-screw-based PDS system has been proved to share loads with the anterior spinal column [7-9,21,22]. According to Wolff’s law, the force transmitted to an interbody graft and avoidance of stress shielding could potentially increase the rate of successful arthrodesis [8,23,24], which was also proved by the biomechanical study of Scifert et al. [25]. Moreover, pedicle-screw-based PDS system standing alone as a non-fusion procedure have been reported to yield satisfactory clinical results, as evaluated by postoperative radiographs and functional scores [10-13]. Without the requirement of bone grafting, soft tissue stripping would not occur beyond bilateral facet joints, blood loss and surgical times would be diminished, and degeneration at the instrumented levels would have the opportunity to be reversed. Reyes-Sa’nchez et al. [10] presented a 2-year clinical report of 20 patients with an AccuFlex system implanted as a stand-alone device for non-fusion procedures. The termination of disc degeneration was observed in 83% of the patients and rehydration was observed in 16% of the patients, despite two cases of implant failure. Regarding the use of a similar system as a preventative reinforcement and an adjunct to fusion surgery, Hudson et al. implanted a rigid fixation system for fusion at the caudal levels, and implanted dynamic instruments at the more cephalic level of the instrumented lumbar vertebrae without fusion in a 28-patient clinical report with 2-year follow-up [26]. No change at the adjacent level observed and disc height was preserved at all levels; furthermore, improved functional outcome was observed. The authors concluded the 2-year study of hybrid dynamic stabilization with a pedicle-screw-based PDS system, which showed satisfactory performance. However, there was no evidence to suggest pedicle-screw-based PDS superior to traditional fusion constructs [13,26,27].

Similarly to the AccuFlex system, the flexibility of the Awesome Dynamic Rod System originates from the cuts created on the surface of the rods. The curved- cut design is similar to that of the AccuFlex, which features helical cuts on the surface of regular rods with the same diameter [21]. The AccuFlex system is approved by the Food and Drug Administration for lumbar fusion when used in conjunction with an anterior interbody device, and the helical cuts on the rods provide flexibility that allows for motion in the flexion-extension mode. According to the biomechanical study conducted by Mandigo et al. [21], the intended flexion and extension movements of the AccuFlex system increase the load transmitted through an anterior interbody graft by more than 50% compared with that afforded by a rigid construct, creating a “load-sharing” capability to increase the potential for fusion through Wolff’s law [23]. Biomechanical tests revealed the rods could withstand the normal stresses exerted on the lumbar spine with an adequate fatigue life. Clinical results indicated that patients to which the AccuFlex system was administered for fusion surgery exhibited statistically similar fusion rates and outcomes compared with patients receiving rigid rod fixation after one year. Another clinical study conducted by Reyes-Sánchez et al. [10] demonstrated that 22.22% of patients receiving AccuFlex constructs for posterior dynamic stabilization required hardware removal due to fatigue, including two cases with broken rods, whereas in 83% of cases no progression of disc degeneration was observed at the instrumented level. Moreover, three patients showed disc rehydration. To prevent cuts from jeopardizing the strength of the rods [10], the Awesome Dynamic Rod System reinforces the segments of rods where cuts are created by enlarging the rod diameter, which are referred to as the “joint parts” of rod; those segments not reinforced are referred to as the “rigid parts” of rods. In addition, unlike the helical cuts of the AccuFlex system, the curve cuts of the Awesome Dynamic Rod System are not helical and allow for the vacant spaces of joint parts to be pressed only in the flexion-extension direction, hindering extra movement of the rods in lateral bending or torsion. However, the effect of enlarging the rod diameter may also neutralize the semi-rigid characteristics provided by the curve cuts.

In the current study, as a posterior dynamic stabilization system, AWE reduced the ROM at the instrumented levels L4-L5 the least in all moments except for extension. As an adjunct to lumbar fusion, AWEFUS reduced the ROM at the instrumented level less than fusion with the rigid rod system, FUS, at all moments except for left bending. Thus, Awesome Dynamic Rod System could preserve motion at the instrumented level better than fusion with rigid rods, not only as a posterior dynamic stabilization system but also as an adjunct to lumbar fusion, although not in all moments. This result is in accord with an in vitro study conducted by Jin et al. [28] comparing the semi-rigid PEEKs rods as a dynamic fixation system, as well as an adjunct to interbody cage fusion procedures, to traditional rigid rods. The authors reported that the PEEK rods sustained lower ROM decrement at the instrumented level than did the traditional rigid rods during flexion, but no significant difference was observed during extension. The curve cuts on the thicker joint parts caused the Awesome Dynamic Rod system to be semi-rigid in the flexion-extension mode but not in lateral bending or in the torsion direction. The Awesome Dynamic Rods did not enhance the dynamic design during extension perhaps due to facet joints withstanding the moment and backward shifting of the helical axis of motion of the lumbar spinal column [29] producing a shorter radius of curvature for a negative bending moment and making it more difficult to bend the dilated joint part of the dynamic rods. Moreover, as an adjunct to lumbar fusion with a cage installed in the disc space, the Awesome Dynamic Rod still demonstrated more flexibility than the rigid rod system did, as indicated by the results obtained for AWEFUS and FUS.

For the adjacent level, i.e., L3-4 for AWE and FUS and L2-3 for AWEFUS, AWE showed a minimal increment, but AWEFUS showed a maximal increment in ROM during flexion, left bending and left torsion. These results suggest that AWE had a weaker effect on the ROM of the adjacent level than did FUS, and potentially alleviating the rate of acceleration of degeneration at the adjacent level. However, to compensate for the maximal decrement in the ROM at the instrumented level during extension, AWE increased the ROM mostly at the adjacent level in the same direction. According to the data presented Table 1, AWEFUS shows a greater increase in ROM at the L1-L2 and L2-L3 level than did the other three models. For AWEFUS, L2-L3 was the level adjacent to the instrumented segments, but the increase in ROM at L2-L3 for AWEFUS was even greater than that at L3-L4 for FUS. However, it is too early to conclude that AWEFUS is almost as stiff as two-level fusion. Using the hybrid method [20] to evaluate the adjacent level effect, the total ROM of the entire spine would be shared by the non-operated levels of the spine models (3 levels for FUS and AWE, and 2 levels for AWEFUS). Hence, observing a much higher ROM for L1-L2 and L2-L3 of AWEFUS would not be surprising. If we summed up the ROM of L1-L2, L2-L3 and L3-L4 of FUS and subtracted less than one degree (the supposed ROM of fused L3-L4) and then divided the result by 2 to simulate the ROM of L1-L2 and L2-L3 at two-level fusion, we would find that the result would still be greater than that for AWEFUS. Thus, when used as a preventative reinforcement, AWEFUS could certainly alleviate the adjacent level effect to a certain extent but not by much. Gillet et al. advocated the application of a type of preventative reinforcement for transitional segments to delay transitional segment alteration after lumbar fusion [14]. Awasthi et al. [16] the presented clinical outcomes of 13 patients treated with the Scient’X Isobar TTL system topping off lumbar fusion. Owing to the distinct improvement in functional scores, including the Oswestry Disability Index and Prolo scale, the authors concluded that the posterior dynamic stabilization device does provide effective stabilization for the transitional segment above a fusion and postpones the rate of degeneration at these cephalic adjacent levels. Hudson et al. [26] prospectively implanted dynamic rod systems at the most cephalic level of lumbar degeneration as a preventative reinforcement on the transitional zone in a clinical study, with fusion performed at the causal levels a of degenerative lumbar spine. The authors did not observe any change in the ROM at the levels above the transitional zone 2 years after operation in compared with that observed upon preoperative measurement. However, the disc height ratio at the level above the index level increased significantly by 14.6%. Nevertheless, the author concluded that the preliminary results at 2 years were satisfactory. As a preventative reinforcement at a level adjacent to that of fusion surgery, AWEFUS did not demonstrate a suitable effect in diminishing the hypermobility of the cephalic adjacent segment, but conversely aggravated its effect on the ROM of the adjacent level, despite providing a sufficient motion constraint at the transitional segment L3-L4.

The same trend was reflected in the disc stress at the adjacent level. That disc stress increases more extensively in flexion than in other moments suggests that discs sustain more stress during flexion. The maximum annulus stress occurring at the anterior edge of the annulus fibrosus during flexion, as shown in Figure 2, corresponds to that reported in a previous study [18], which also demonstrated that the greatest annulus stress occurred at the adjacent cranial level located at the anterior edge of the annulus fibrosus. The disc stress distribution shown in Figure 2 reveals that AWE showed a smaller increase in stress at the adjacent level than did FUS. This finding corresponds well with the results obtained for ROM at the adjacent level and suggests that AWE showed less stress concentration than did FUS at the adjacent level. Disc stress at L3-4 of AWEFUS was shielded by the Awesome Dynamic Rod System. As was observe for ROM, however, the disc stress at the L2-L3 level of AWEFUS was much greater than that observed for the other three models. AWEFUS did not eliminate stress effectively at the adjacent disc when acting as a preventative reinforcement in the transitional zone.

The maximal facet contact forces of INT at the L4-L5 level were 135 N in torsion, 54 N in extension, and 21 N in lateral bending. There was no contact force in flexion. This result is in agreement with the FE study conducted by Wilke et al., who loaded a pure 7.5 Nm stress on a L4-L5 FE spinal model. The authors predicted maximum facet joint forces of 105 N under torsion, 50 N under extension and 36 N under lateral bending, and the facet joints remained unloaded during flexion [30]. At the adjacent level, AWE showed a lower contact force than did FUS in left bending and left torsion but a greater contact force in extension. As demonstrated in a previous study [17,18], the incremental increases in the facet joint contact forces at the cranial adjacent level were proportional to the segment stiffness at the instrumented level.

The limitations of the FE analysis conducted in this study are as follows. The characteristics of disc degeneration, such as dehydration and reduced disc height, were not taken into account. The thread on the pedicle screw was ignored. Because this study aimed to examine how the new Awesome Rod System affects spinal biomechanics rather than the mechanical interaction between screw threads and bone, this study assumed complete osteointegration in between bone and screw. The conclusions drawn in this study are based on these limitations. Additionally, the FE study could only demonstrate the biomechanical effects of the implants when moments were loaded on the models. Further clinical studies should be conducted to monitor long-term implant fatigue and graft fusion.

## Conclusion

In view of results of this study, used as a stand-alone dynamic fixation, Awesome Dynamic Rod System could preserve more instrumented segment motion than traditional rigid fixation. Used at an adjunct to fusion procedure, Awesome Dynamic Rod System could provide more instrumented segment motion than traditional rigid fixation and augmenting interbody graft fusion. Used as a preventative reinforcement for fusion surgery, Awesome Dynamic Rod System did protect the segment adjacent to fusion segments, but led much ROM, disc stresses, and facet joint contact forces increasing at the segment adjacent to instrumented segments.
